# Correlations between Phytohormones and Drought Tolerance in Selected *Brassica* Crops: Chinese Cabbage, White Cabbage and Kale

**DOI:** 10.3390/ijms19102866

**Published:** 2018-09-21

**Authors:** Iva Pavlović, Ivan Petřík, Danuše Tarkowská, Hrvoje Lepeduš, Valerija Vujčić Bok, Sandra Radić Brkanac, Ondřej Novák, Branka Salopek-Sondi

**Affiliations:** 1 Laboratory for Chemical Biology, Department of Molecular Biology, Ruđer Bošković Institute, Bijenička cesta 54, Zagreb 10000, Croatia; iva.pavlovic@irb.hr; 2 Laboratory of Growth Regulators, Centre of the Region Haná for Biotechnological and Agricultural Research, Faculty of Science of Palacký University & Institute of Experimental Botany of the Czech Academy of Sciences, Šlechtitelů 27, Olomouc 78371, Czech Republic; petrik@ueb.cas.cz (I.P.); tarkowska@ueb.cas.cz (D.T.); 3 Faculty of Humanities and Social Sciences, and Faculty of Dental Medicine and Health, Josip Juraj Strossmayer University of Osijek, Lorenza Jägera 9, Osijek 31000, Croatia; hlepedus@yahoo.com; 4 Department of Biology, Faculty of Science, University of Zagreb, Rooseveltov trg 6, Zagreb 10000, Croatia; vvujcic@biol.pmf.hr (V.V.B.); sandra@biol.pmf.hr (S.R.B.)

**Keywords:** *Brassica* crops, phytohormones, drought, recovery, tolerance

## Abstract

Drought is one of the major abiotic stresses affecting the productivity of *Brassica* crops. To understand the role of phytohormones in drought tolerance, we subjected Chinese cabbage (*B. rapa* ssp. *pekinensis*), white cabbage (*B. oleracea* var. *capitata*), and kale (*B. oleracea* var. *acephala*) to drought and examined the stress response on the physiological, biochemical and hormonal levels. The phytohormones abscisic acid (ABA), auxin indole-3-acetic acid (IAA), brassinosteroids (BRs), cytokinins (CKs), jasmonates (JAs), and salicylic acid (SA) were analyzed by ultra-high-performance liquid chromatography–tandem mass spectrometry (UHPLC-MS/MS). Based on the physiological and biochemical markers the Chinese cabbage exhibited the lowest tolerance, followed by the white cabbage, while the kale appeared to be the most tolerant to drought. The drought tolerance of the kale correlated with increased levels of SA, ABA, IAA, CKs iP(R) and *c*Z(R), and typhasterol (TY), a precursor of active BRs. In contrast, the drought sensitivity of the Chinese cabbage correlated with a significant increase in ABA, JAs and the active BRs castasterol (CS) and brassinolide (BL). The moderately tolerant white cabbage, positioned between the kale and Chinese cabbage, showed more similarity in terms of the phytohormone patterns with the kale. We concluded that the drought tolerance in Brassicaceae is mostly determined by the increased endogenous levels of IAA, CKs, ABA and SA and the decreased levels of active BRs.

## 1. Introduction

*Brassica* vegetables, belonging to the family Brasiccaceae, include many economically important species that are grown worldwide. The most utilized *Brassica* vegetables are *B. oleracea* and *B. rapa,* comprising different headed and non-headed cabbages, kale, broccoli, cauliflower, and Brussels sprouts. Around 70 million tons of cabbages are produced worldwide every year [1], and about 90% of the world’s cabbage is produced in Europe and Asia.

Cabbage production is strongly influenced by environmental conditions. In light of the current changes observed in the climate, drought is considered to be one of the most serious problems for global agriculture, affecting approximately 40% of the world’s land area [2,3]. Since *Brassica* crops are mainly cultivated commercially in Mediterranean, semi-arid and arid environments, their growth, and consequently the crop yield and quality, can be greatly impaired by drought [3]. Future projections are even worse, since a gradual, but consistent, increase in aridity and drought is estimated for most of the Mediterranean region [4].

Plant response to drought represents a complex network of cellular and molecular processes, which result in the establishment of novel homeostasis in order to ensure survival under unfavorable environmental conditions. The roots are the first organs exposed to water deficiency in the soil and are the location of drought sensing. Chemical signals from the root are transported towards the shoot and initiate molecular and biochemical processes, which finally moderate the morphological responses, enabling plants to cope with the drought conditions [5]. Drought stress-responsive genes are thought to be regulated through complex regulatory networks of kinases, transcription factors, reactive oxygen species (ROS) and phytohormones. The cellular effects of drought stress mostly include imbalances of osmotic homeostasis, but also impaired photosynthesis, cellular energy depletion, and redox imbalances [6]. These mechanisms rely on a complex cellular metabolism, in which almost all known phytohormones play an important role [7,8]. In addition, recent investigations have shown that phytohormones (such as ABA, auxins, CKs, SA, JAs, BRs, etc.) have the potential to enhance the abiotic stress tolerance (including drought tolerance) of various plant species, and may be considered an important metabolic target for producing abiotic stress-tolerant crop plants [9,10].

When considering crops from the *Brassica* genus and their abiotic stress responses, the most investigated species is the Chinese cabbage (*B. rapa*). Recent genome-wide analyses from *B. rapa* have suggested that various plant hormones are involved in the drought stress responses, although systematic phytohormone profiling has not been carried out so far [11,12,13]. Next-generation sequencing of *B. juncea* seedlings subjected to high temperature and drought stresses deciphered the genome-wide perturbations of steady-state levels of transcripts [14]. Approximately 19,000 transcripts were found to be differentially regulated upon stress treatments, and among them, many transcripts were related to hormone signaling (auxin, SA, JA, ABA, and gibberellins). Other economically important *Brassica* crop species, such as white cabbage and kale, have only been sporadically monitored [3]. Comparative mapping between *Brassica* and *Arabidopsis* with the SSR marker is helpful to identify candidate genes for tolerance against abiotic stresses, such as salinity stress, etc. [15].

However, the lack of systematic hormonomic research and elucidation of the role of phytohormone in the drought response and the regulation of drought tolerance in *Brassica* crops is evident. Our hypothesis is that certain plant hormones are responsible, in part, for the drought tolerance in *Brassica* crops. Thus, our research includes an analysis of several groups of phytohormones (ABA, auxins, CKs, JAs, SA, and BRs) in selected *Brassica* varieties (*B. rapa*, *B. oleracea* var. *capitata* and *B. oleracea* var. *acephala*) that exhibit a different sensitivity/tolerance to drought stress. Biochemical and physiological stress markers were measured in parallel with a hormonal analysis. The resulting hormonal profiles are discussed in relation to the plants’ physiological (relative water content (RWC) and photosynthesis rate) and biochemical stress markers (malondialdehyde (MDA), protein oxidation, proline, and antioxidant enzymes).

## 2. Results

### 2.1. Physiological and Biochemical Responses of Brassica Crops to Drought and Recovery

**Figure 1:**
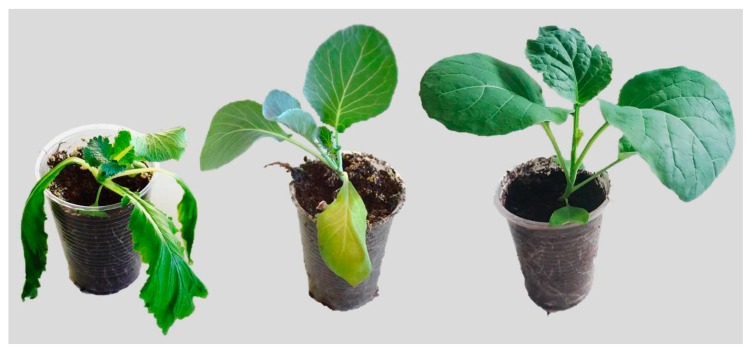
*Brassica rapa* (**left**), *B. oleracea* var. *capitata* (**middle**), and *B. oleracea* var. *acephala* (**right**) after 7 days of water deprivation.

Three *Brassica* cultivars—Chinese cabbage (*B. rapa)*, white cabbage (*B. oleracea* var. *capitata)*, and non-headed kale or black cabbage (*B. oleracea* var. *acephala*)—were investigated under drought stress and recovery. Each of these cultivars reached a RWC of 45 ± 10% following different times of water deprivation: the Chinese cabbage after approximately 7 days, the white cabbage after approximately 10 days, and the kale after approximately 15 days. During the period of drought, the plants were inhibited in growth and showed clear signs of dehydration, particularly *B. rapa,* in comparison to the *B. oleracea* varieties (var. *capitata* and var. *acephala*). Figure 1 shows the phenotype of the *Brassica* crops *B. rapa*, *B. oleracea* var. *capitata* and *B. olerace* var. *acephala* after 7 days of water deprivation. While the sensitive *B. rapa* shows clear signs of dehydration, the white cabbage and kale are healthier, with only slight signs of dehydration in the white cabbage and no change in the kale. Following 24 h of recovery, the RWC values were 86%, 80%, and 86% in the Chinese cabbage, white cabbage and kale, respectively.

**Figure 2:**
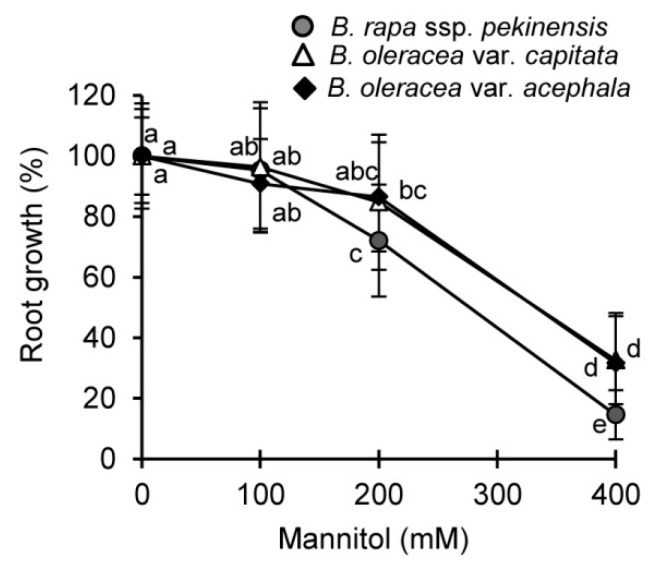
Root-growth assay of *Brassica* seedlings treated with mannitol (0–400 mM). Different letters present significant values (*p* ≤ 0.05). Data are mean ± SD, *n* = 30–33.

In addition, we evaluated the drought tolerance of selected *Brassica* crops in vitro, on the level of one-day-old seedlings, by root-growth assay using mannitol (0–400 mM) as an osmotic stressor (Figure 2). It is clear that the seedlings of *B. rapa* were strongly affected by the increasing concentration of mannitol in comparison to the *B. olerace*a varieties. At the highest mannitol concentration (400 mM) root-growth was 15%, 33% and 32% of controls in the *B. rapa*, *B. oleracea* var. *capitata* and *B. oleracea* var. *acephala*, respectively.

To detect the influence of drought and recovery on the photosynthesis of the *Brassica* crops, the chlorophyll *a* fluorescence was measured in vivo and selected parameters, i.e., the performance index (PI_ABS_) and maximum quantum yield of PS II (F_v_/F_m_), are presented in Table 1. Under our experimental conditions PI_ABS_ was significantly decreased in the Chinese and white cabbage during drought (by approximately 50%), while this parameter did not statistically change in the kale. After re-watering, the PI_ABS_ recovered completely in the Chinese cabbage and showed a tendency to increase, reaching the level of the control in the white cabbage. In the kale, the PI_ABS_ was not significantly changed after recovery in comparison with the control and the plants in drought. The F_v_/F_m_ parameter was less affected by drought than the PI_ABS_. It was significantly decreased only in the *B. rapa* under drought (11%, regardless of the control).

**Figure 3:**
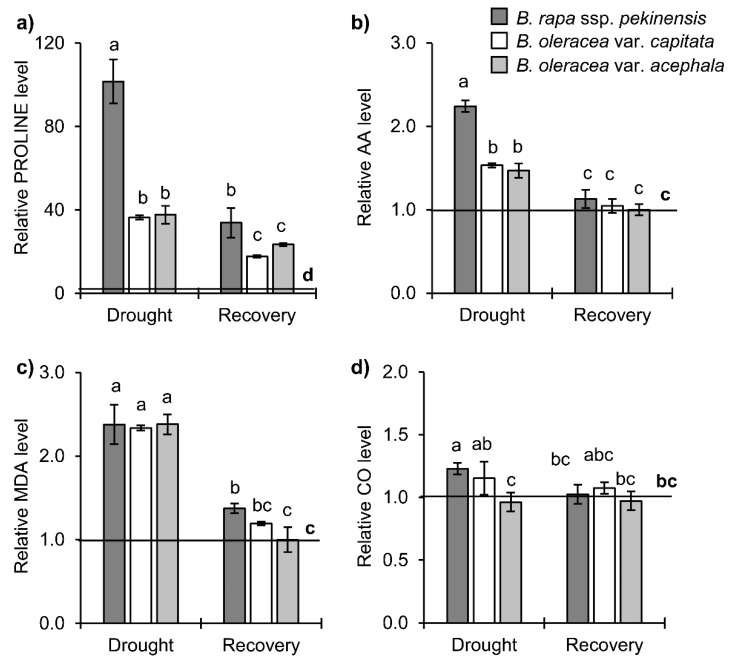
The contents of (**a**) proline, (**b**) ascorbic acid (AA), (**c**) malondialdehyde (MDA) and (**d**) reactive carbonyl groups (CO) in the cabbage leaves after the drought period (at the point when the RWC was 40–50%) and 24 h after re-watering. Controls are normalized to the value 1. Columns marked with different letters are statistically significantly different (*p* ≤ 0.05). The bars represent mean ± SD, *n* = 4. Raw data are presented in Supplementary Table S2.

Biochemical markers of water stress clearly showed that all three *Brassica* crops exhibited significantly increased levels of MDA, proline, and AA under drought, regardless of the corresponding controls (Figure 3). Proline appeared to increase the most remarkably: 102-fold in the Chinese cabbage, 38-fold in the white cabbage and 36-fold in the kale. It is interesting to note that the white cabbage and the kale contain approximately 3 times more proline in the control plants in comparison to the Chinese cabbage (see original data in Supplementary Table S2). The level of protein oxidation estimated as reactive carbonyl groups increased significantly only in *B. rapa* upon drought, while *B. oleracea* var. *capitata* and *B. oleracea* var. *acephala* did not show differences in comparison to the controls. After the period of drought, the plants were re-watered overnight. In the plants recovered for 24 h, the biochemical parameters returned to the level of the controls (AA, reactive carbonyl groups and MDA) or showed a decreasing pattern towards the level of the controls, as was the case with proline (Figure 3).

**Figure 4:**
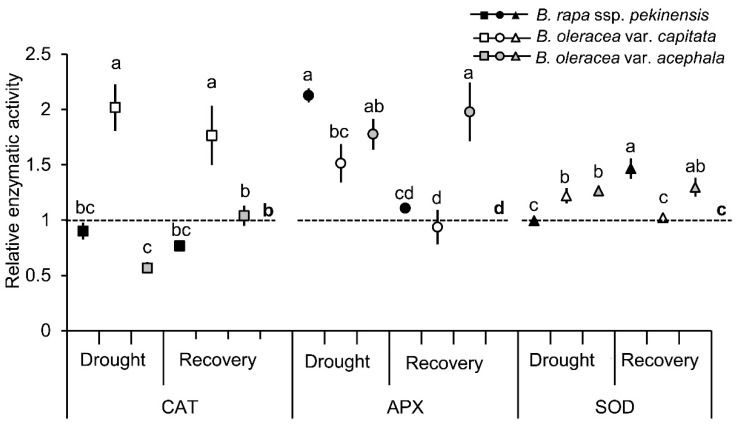
Antioxidant enzyme assays: catalase (CAT), ascorbate peroxidase (APX) and superoxide dismutase (SOD) in leaves of *Brassica* crops under drought period and following recovery. Controls are normalized to the value 1 (broken line). The bars are mean ± SD, *n* = 4. Points marked with different letters differ significantly at *p* < 0.05. Raw data are presented in Supplementary Table S3.

Among the biochemical stress markers, we measured the anti-oxidant activities of SOD, CAT and APX in the selected cultivars under stress conditions (Figure 4). The pattern of CAT activity was different in all three cultivars during drought and recovery. There was a significant increase in the CAT activity in the white cabbage, a significant decrease in the kale, and there was no difference in the Chinese cabbage under drought. After re-watering, the CAT activity in the Chinese cabbage remained high, while that in the white cabbage and the kale return to the control level. The APX activity was significantly increased upon drought in all the examined cultivars, regardless of the corresponding controls. After re-watering, the APX activity decreased to the level of the controls in the white and Chinese cabbage, while it remained significantly high in the kale leaves. The SOD activity was significantly increased in the white cabbage and the kale under drought in comparison to the controls, while it did not change significantly in the *B. rapa*. Following re-watering, the SOD activity of the white cabbage decreased to the control level, while that of the Chinese cabbage and kale increased significantly compared to their corresponding controls.

### 2.2. Stress-Related Phytohormone Profiles in Brassica Crops under Drought and Recovery 

**Figure 5:**
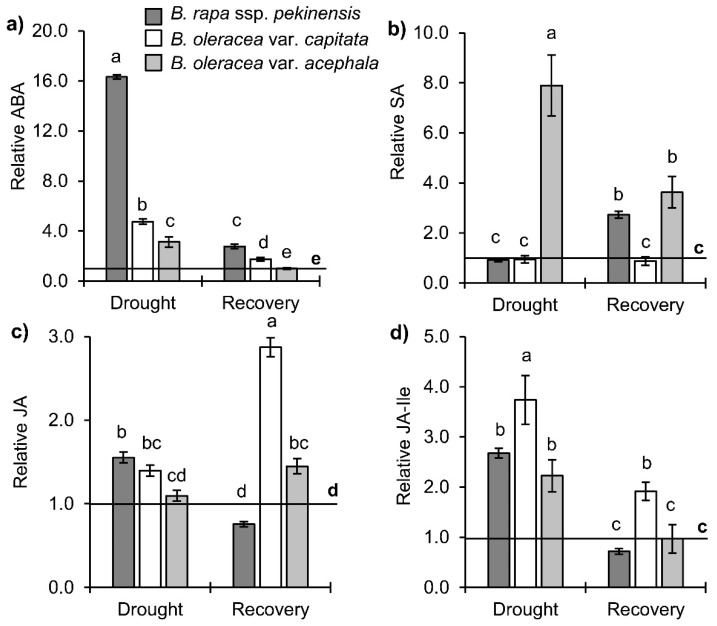
Stress hormones profile in *Brassica* crops under drought and recovery: (**a**) ABA, (**b**) SA and jasmonates (**c**) JA and (**d**) JA-Ile. Controls are normalized to the value 1. Columns marked with different letters are statistically significantly different (*p* ≤ 0.05). Data are mean ± SD, *n* = 4. Raw data are presented in Supplementary Table S4.

ABA, SA, and jasmonates (JA and JA-Ile) were detected in three *Brassica* cultivars under drought stress and following recovery (Figure 5). ABA was significantly increased under drought in all three *Brassica* varieties. The largest increase was obtained in *B. rapa* (16.3-fold), then in *B. oleracea* var. *capitata* (4.8-fold) and *B. oleracea* var. *acephala* (3.1-fold). The level of ABA decreased in all three cultivars after recovery. The SA content remained unchanged under stress and recovery conditions in the white cabbage. A significant increase in SA content was obtained in the *B. oleracea* var. *acephala* under drought (7.9-fold), and it decreased twice after recovery. Furthermore, drought did not influence the level of SA in the *B. rapa*, but recovery significantly increased the level of SA, regardless of the control and the drought plants (approximately 2.7-fold). Drought conditions caused a significant increase of JA in the Chinese (1.6-fold) and white (1.4-fold) cabbages (Figure 5c). JA-Ile was significantly increased in all three cultivars, particularly in the white cabbage (3.7-fold) (Figure 5d). Recovery caused an additional increase of JA in the white cabbage, while the JA content returned to the level of the control in the kale or even decreased to below the control level in the Chinese cabbage upon re-watering. The JA-Ile content was in agreement with JA fluctuations in the three *Brassica* crops upon recovery (Figure 5c,d).

### 2.3. Brassinosteroids in Brassica Crops under Drought and Recovery

**Figure 6:**
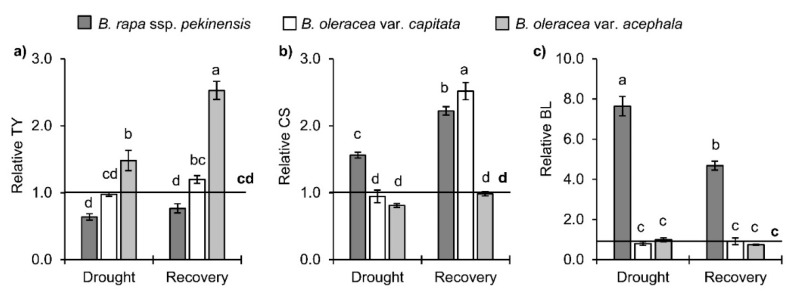
Brassinosteroids (**a**) typhasterol (TY), (**b**) castasterone (CS) and (**c**) brassinolide (BL) in *Brassica* crops under drought and recovery. Controls are normalized to the value 1. Columns marked with different letters are statistically significantly different (*p* ≤ 0.05). Data are mean ± SD, *n* = 3. Raw data are presented in Supplementary Table S5.

Three brassinosteroids, i.e., typhasterol (TY), castasterone (CS) and brassinolide (BL), were found and quantified in the *Brassica* crops upon drought and recovery (Figure 6a–c). It is clear that the drought caused only a significant increase of TY in the kale (48%), and did not change significantly the level of TY in the white and Chinese cabbages in comparison to the control. Upon recovery, the TY level was additionally increased in the kale (1.7-fold in comparison to the drought). In contrast to the TY in drought conditions, the CS increased in the *B. rapa* (1.6-fold), while the CS level did not change in the white cabbage and the kale in comparison with the controls. Upon recovery, the CS was significantly increased in the Chinese and white cabbages (2.2 and 2.5-fold, respectively), while it was at the level of the control in the kale. The BL was significantly increased in the *B. rapa* under drought (7.6-fold), regardless of the control. After re-watering, the BL was approximately halved in the *B. rapa*, but it was still significantly higher than in the control. The BL was not influenced by the drought conditions and recovery in the white cabbage and the kale.

### 2.4. Auxin Levels in Drought and Recovery

**Figure 7:**
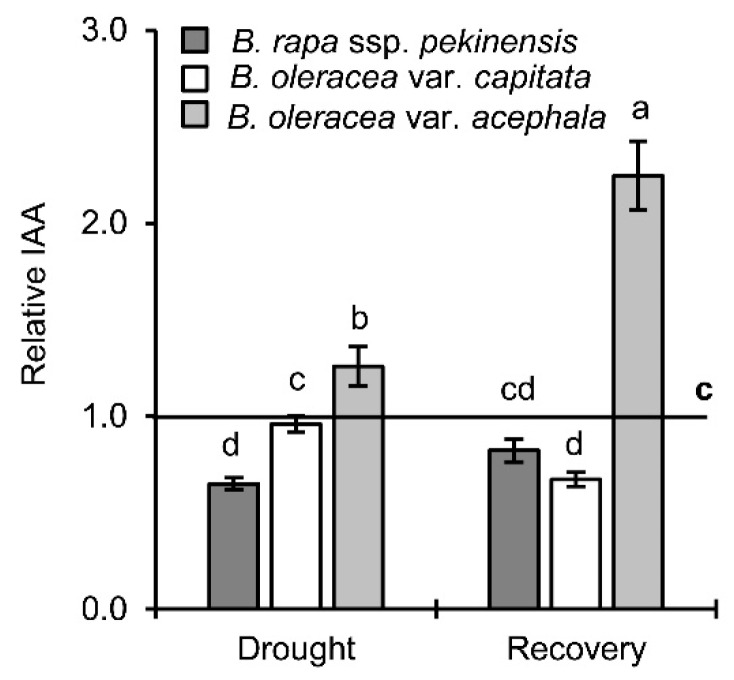
Level of indole-3-acetic acid (IAA) in *Brassica* crops under drought and recovery. Controls are normalized to the value 1. Columns marked with different letters are statistically significantly different (*p* ≤ 0.05). Data are mean ± SD, *n* = 4. Raw data are presented in Supplementary Table S6.

As can be seen in Figure 7, a significant decrease of IAA was obtained in the *B. rapa* upon drought (35%), while it increased in the *B. oleracea* var. *acephala* (26%), regardless of the controls. Re-watering caused an additional significant increase of IAA in the kale (2.3-fold in comparison to the control), and a decrease in the white cabbage. In the Chinese cabbage the IAA increased in comparison to the drought, and reached the level of the control.

### 2.5. Cytokinin Profile in Drought and Recovery

**Figure 8:**
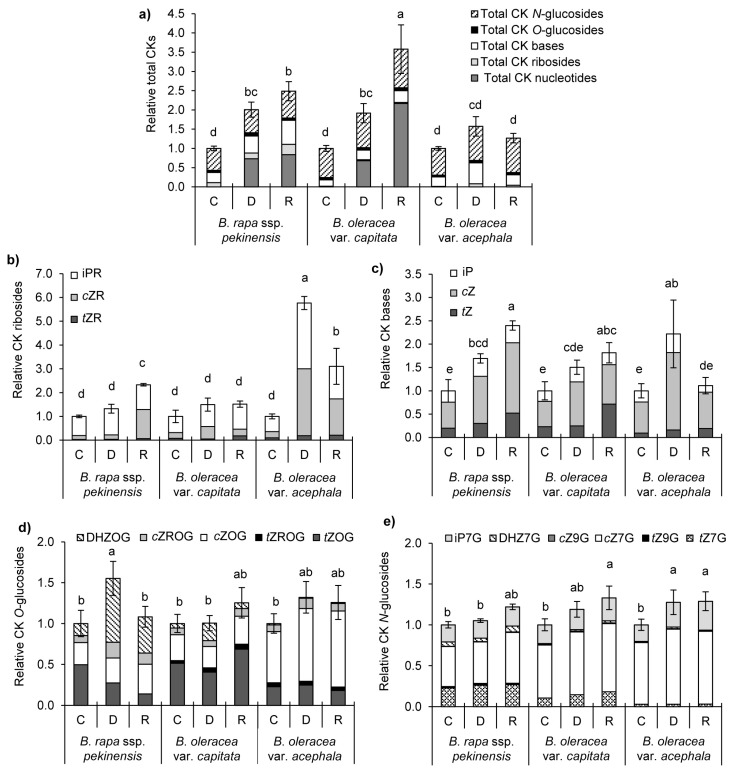
Cytokinins profile in *Brassica* crops (*B. rap*a, *B. oleracea* var. *capitata*, and *B. oleracea* var. *acephala*) under drought (**D**) and recovery (**R**) in comparison to the corresponding controls (**C**); (**a**) Fluctuation of total cytokinins and distribution of individual CKs groups (nucleotides, ribosides, bases, *O*-glucosides, and *N*-glucosides); data are mean of 4 replicates; error bars present SD of total CKs. Fluctuations of CKs groups and individual types of CKs among group: (**b**) ribosides (R), (**c**), active bases (*trans-*zeatin, *t*Z; *cis-*zeatin, *c*Z; dihydrozeatin, DHZ; isopentenyladenine, iP), (**d**) *O*-glucosides (OG), and (**e**) *N*-glucosides (7G and 9G). Controls are normalized to the value 1. Columns marked with different letters are statistically significantly different (*p* ≤ 0.05). Data are means of 4 replicates; error bars present SD of total CKs groups. Raw data, normalized data and statistical analysis of individual CKs are given in Supplementary Tables S6–S9.

Isoprenoid CK metabolites (ISCK), representing nucleotides (precursors), ribosides (transported forms), free bases (active forms), and glucosides (storage forms) of *trans-* and *cis-*zeatin (*t*Z and *c*Z), dihydrozeatin (DHZ), and isopentenyladenine (iP)-types, were found upon drought and recovery in selected *Brassica* crops (Figure 8). Under our experimental conditions, the total CK levels were significantly increased in the drought-stressed Chinese and white cabbages (2.0 and 1.9 times, respectively), while they did not change significantly in the kale, in comparison to their controls (Figure 8a). An additional increase in the total ISCK was observed after recovery in the Chinese and white cabbages (2.5- and 3.6-fold, respectively) in comparison to the controls. Moreover, the profile of particular CK groups and the abundance of their individual CK types are shown in Figure 8b–e.

Based on our findings, the CK biosynthesis was greatly altered, including the CK nucleotides, ribosides and bases. No CK nucleotides were detected in the samples, except for the *cis*-zeatin riboside-5’-monophosphate (*c*ZRMP), which was significantly increased in the *B. rapa* and *B. oleracea* var. *capitata* under drought and recovery conditions (see Supplementary Table S7). Interestingly, the presence of *c*ZRMP was not confirmed in the kale. An additional increase of this precursor was obtained after recovery, to 104.4 and 313.1 pmol DW^−1^, in the Chinese and white cabbages, respectively. Accordingly, a significant increase in the total CKs in the Chinese and white cabbages upon drought and recovery were a result of the significant increase in CK precursors. 

The fluctuation of CK ribosides under drought and recovery in the investigated *Brassica* crops is shown in Figure 8b. A significant increase in the total riboside was measured only in the kale upon drought, with a particularly significant increase in isopentenyladenosine (iPR) and *cis*-zeatin riboside (*c*ZR) in comparison to the control. Following recovery, the level of ribosides was significantly high in the *B. rapa* and *B. oleracea* var. *acephala*, regardless of the controls. 

The total active CKs increased by 1.7-, 1.5- and 2.2-fold in the Chinese cabbage, white cabbage and kale, respectively, under drought (Figure 8c). An additional increase in the total free CK bases (2.4-fold in comparison to the control) was only found in the *B. rapa* after recovery. The most prominent change appeared in the level of *c*Z, which approximately doubled in the Chinese and white cabbages, and increased 2.5-fold in the kale, upon drought ( Supplementary Table S9). Following recovery, the *c*Z only remained high in the *B. rapa* (3-fold in comparison to the control). 

The total *O*-glucosides, reversibly inactivated storage forms, increased significantly only in the *B. rapa* upon drought, and it was mostly based on the increase of DHZOG (Figure 8d). Among the total *N*-glucosides, irreversible inactivated storage forms, mostly levels of isopentenyladenine 7-glucoside (iP7G) and *cis*-zeatin 7-glucoside (cZ7G), increased significantly in the kale upon drought, and their levels were significantly high in the white cabbage and the kale in comparison with the corresponding controls after recovery (Figure 8e).

### 2.6. Principal Component Analysis (PCA)

**Figure 9:**
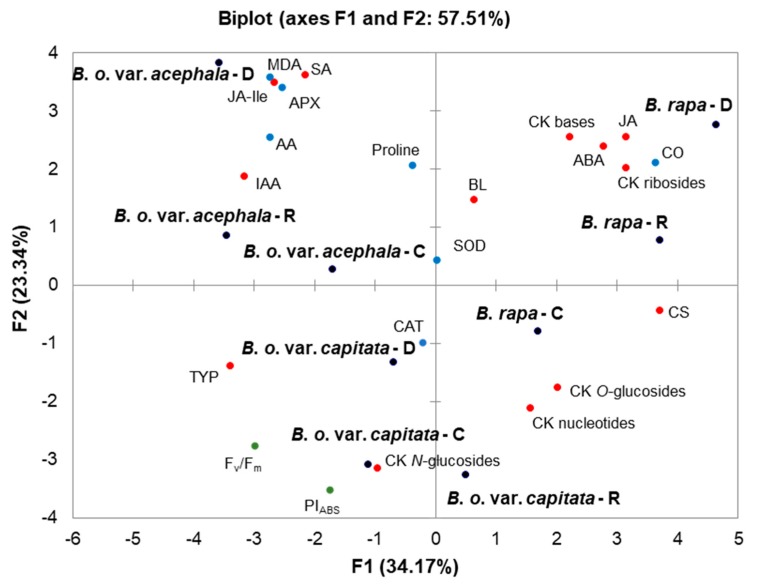
The two-dimensional principal component analysis (2D-PCA) of drought and recovery of *B. rapa*, *B. oleracea* var. *capitata*, and *B. oleracea* var. *acephala*, and resulting biochemical and hormonal responses. PCA was performed on the correlation matrix of standardized average values of biochemical parameters (Table 1, Figure 3 and Figure 4), and phytohormone profiles (Figure 5, Figure 6, Figure 7 and Figure 8). Cytokinins are presented as total CK groups. Green, blue and red dots present photosynthetic parameters, biochemical markers, and phytohormones, respectively. Treatments: drought (**D**), recovery (**R**) and the control (**C**). Correlation matrix data, eigenvalues, factor loadings and factor scores are given in Supplementary Tables S10–S14.

To clarify the relations among the measured parameters, a PCA was performed on the whole set of standardized average values of the biochemical stress markers and phytohormones, upon drought, recovery and the corresponding controls (Figure 9). Pearson linear coefficients and the probability of the correlations (at *p* < 0.05) were calculated, and the results are presented in Supplementary Table S10. Figure 9 shows two-dimensional plots, which demonstrate: (i) the positioning of the *Brassica* crops considering the treatments and controls relative to each other; and (ii) the relations among the obtained biochemical and hormonal parameters upon treatments (drought and recovery, regardless of the controls). The principle components F1 and F2 describe 34.17% and 23.34% of the original information, explaining 57.51% of the total variability among the treatments for all the traits investigated (Supplementary Table S11). To investigate the contributors to the principle component, the loadings in F1 and F2 were compared (Supplementary Tables S12 and S13). Accordingly, in drought conditions, the *Brassica* varieties were positioned far away from each other, suggesting their different responses, and consequently their different tolerances to water deficit. Their controls were situated closer to each other than the samples in drought, while following recovery all the varieties were positioned on the plot between the drought and the controls with corresponding loadings (Supplementary Table S13). 

Furthermore, Figure 9 illustrates the relationships among the particular parameters in this study. The contribution of a particular parameter to the principle components can be seen in Supplementary Table S14. The stress hormone SA was positioned close to the *B. oleracea* var. *acephala* upon drought, while the ABA was positioned closer to the *B. rapa* variety. Jasmonates were situated quite differently, suggesting that JA was changed mostly in the *B. rapa* in drought, while the most prominent change of the JA-Ile level was obtained in the *B. oleracea* varieties. IAA was, on the other hand, positioned close to the *acephala* variety, indicating the most prominent change in the kale upon drought. Based on the Pearson coefficient, SA and JA-Ile were positively correlated with the biochemical markers MDA and APX, as well as each other, while ABA and JA were negatively correlated with the photosynthetic parameters (PI_ABS_ and F_v_/F_m_) and positively correlated with the CO and each other (Supplementary Table S10). Considering brassinosteroids, it was noted that the TY was grouped with the *B. oleracea* varieties. On the other hand, the CS and BL were positioned closer to *B. rapa*. Cytokinins were shown as total CK groups. It is clear that the nucleotides, *O*-glucosides and *N*-glucosides were grouped together with a negative loading on F2. Based on the Pearson coefficient, the nucleotides are positively correlated with the *O*- and *N*-glucosides (*n* = 0.7427, and *n* = 0.6885, respectively). On the other hand, ribosides and free bases were positioned together and they positively correlated with each other (*n* = 0.8376). It was also noted that the free bases and ribosides positively correlated with the brassinosteroid CS (*n* = 0.7039, and *n* = 0.7865, respectively). In addition, the CK ribosides significantly negatively correlated with the brassinosteroid TY (*n* = −0.7134) (Supplementary Table S10).

## 3. Discussion

Drought stress is one of the most severe abiotic stresses, and can affect the growth, yields, and product quality of *Brassica* crops, particularly in Mediterranean, semi-arid and arid environments. Depending of their tolerance, plants have developed different mechanisms to cope with drought at the morphological, physiological, cellular and molecular levels [16]. These mechanisms are regulated by complex networks of phytohormones actions. So-called stress hormones, i.e., ABA, SA, JAs and ET, are well known for their regulatory response in drought stress as key plant hormones. Recently, auxins, cytokinins, gibberellins, brassinosteroids and strigolactones have also been reported to affect the drought-signaling cascade and could be integrated with stress hormones for better plant survival [8]. There are sporadic reports on the correlations of drought tolerance and the phytohormones level, but there are no clear and consistent conclusions [7]. To shed light on this issue for Brassicaceae, we investigated the impact of water deprivation on three *Brassica* crops, widely grown worldwide, that exhibit different drought tolerances, with a particular focus on their phytohormone responses. 

### 3.1. Selected Brassica Crops Exhibit Different Sensitivity/Tolerance to Drought: Physiological and Biochemical Stress Markers 

The first parameter indicating the different drought tolerances among the selected *Brassica* crops was the different periods of water deprivation necessary to reach 55–45% of RWC in the treated plants: approximately 7 days for *B. rapa*, 10 days for *B. oleracea* var. *capitata* and 15 days for *B. oleracea* var. *acephala*. Accordingly, *B. rapa* was suggested as being the most sensitive, followed by *B. oleracea* var. *capitata*, and *B. oleracea* var. *acephala* as the most tolerant variety. A root-growth bioassay also confirmed the same trend of drought tolerance among the selected crops. Thus, we measured the photosynthetic and biochemical parameters in the treated plants, regardless of the controls, to learn more about the stress response of the selected species. Typical plants’ photosynthetic reaction to water deficit is triggered by a stomatal closure that, in turn, disturbs the photosynthetic electron transport and leads to photo damage that occurs at the PS II reaction center and the oxygen-evolving complex (OEC). This, consequently, causes excessive production of the ROS [17]. 

Two frequently used photosynthetic parameters that serve as stress indicators in plants are the maximum quantum yield of PS II (F_v_/F_m_) and the performance index (PI_ABS_), both determined by measuring chlorophyll *a* fluorescence that originates almost exclusively from PS II. As revealed by previous investigations, the F_v_/F_m_ parameter was not affected until severe drought stress occurred, while the PI_ABS_ appeared to be much more sensitive to drought stress [18,19,20]. This corresponded to our results shown in Table 1. The drought-sensitive *Brassica* species (the Chinese and white cabbages) revealed a much more pronounced decrease of PI_ABS_ than F_v_/F_m_. The observed discrepancies between the dynamics of the two investigated photosynthetic parameters indicate that the primary photochemistry was not disturbed by the drought stress, but by the electron transport [21,22]. Since the kale showed no decrease for both the investigated photosynthetic parameters (Table 1), it can be considered an extremely drought-tolerant species. Furthermore, the photosynthetic responses of the drought-sensitive *Brassica* species revealed a tendency to re-establish competent photosynthetic performance during the recovery period (Table 1). That would be in agreement with the mechanisms described by Goltsev et al. [21], where drought stress between 60 and 20% of RWC suppressed the electron transport from the PQ pool to the PS I, which seems to be an easily reversible drought-stress consequence of re-watering, without a disturbance of the PS II primary photochemistry.

Among the biochemical stress makers, drought tolerance is often associated with the accumulation of proline and an efficient anti-oxidative system, which can prevent the damage induced by the enhanced formation of ROS. Upon drought conditions, the proline accumulated at much higher levels in comparison to the respective controls in the examined *Brassica* cultivars, suggesting an osmoregulatory role of that metabolite. The proline protective mechanisms have also been proposed to involve the stabilization of anti-oxidant enzymes (especially the activities of enzymes in the ASC-GSH cycle), as well as direct scavenging of the ROS and a consequent decrease of the lipid peroxidation [23]. In this study, malondialdehyde, an indicator of lipid peroxidation, increased in the *Brassica* cultivars, thus excluding the potential role of the proline in the detoxification of the ROS. On the other hand, proline accumulation coincided with the upregulation of the APX activity in the *Brassica* crops, which might be connected with the stabilizing effects of proline. Similar results (the prooxidant action of proline manifested as the accumulation of MDA and the induction in SOD and especially APX activities) have already been reported [24].

Regarding the changes in the anti-oxidative enzyme, the activities of SOD and APX were increased or unaffected by drought, while the CAT activity decreased in all the *Brassica* cultivars, except in the Chinese cabbage. The decline in CAT activity might be due to the inhibition of the enzyme synthesis or a change in the assembly of enzyme subunits under stress conditions. Catalase can also be a target of the peroxisomal protease activity [25]. Thus, the induction of APX might be considered a key point for the decomposition of H_2_O_2_ in *Brassica* cultivars. Concerning the differential response of *Brassica* cultivars to drought, it was interesting to note that the kale exhibited higher constitutive and drought-induced levels of ascorbate, APX and CAT, as well as the enhanced activity of SOD under drought. Such results, together with the results of photochemical efficiency, suggest better drought-tolerance mechanisms in the kale compared with the Chinese and white cabbage.

### 3.2. Hormonal Profile upon Drought and Recovery in Investigated Brassica Crops

#### 3.2.1. Stress Hormones

The stress hormones ABA, JA and SA have been implicated as key players in drought-stress signaling, based on their accumulation during drought and their positive regulatory role in drought tolerance [8]. ABA has been reported to be a pivotal regulator in the activation of a plant’s adaptation to drought and has an important role as a growth inhibitor [6]. ABA is known to regulate many biochemical processes, such as proline accumulation and other anti-oxidants as well as anti-oxidant enzymes’ activities, in order to cope with an unfriendly environment [26]. ABA is the main regulator of photosynthesis, since it regulates the stomatal closure in conditions of water deficit and causes a decrease in the photosynthetic performance, as we identified in drought-sensitive *B. rapa*. It was shown that mutants having non-functional ABA biosynthesis are particularly sensitive to water deficit, with the exception of transgenic plants, with an increased ABA level that helps with survival in an adverse environment [27]. It was also shown that exogenously applied ABA effectively improved the drought tolerance in creeping bentgrass (*Agrostis stolonifera*) by maintaining membrane stability and leaf-water status [28]. Our results showed a significant increase in the ABA level for all three Brassicas upon drought, in comparison with their controls, which is in agreement with the published data [7,27]. However, ABA accumulation in *B. rapa* was 3.4 and 5.2 times greater than in the white cabbage and kale during drought, respectively. The correlation between ABA’s endogenous level and the stress tolerance is not unambiguous in the plant kingdom. The endogenous level of this hormone oscillates according to its metabolism and transport through the plant, plant species and organ/tissues, as well as the duration and severity of the drought stress. There are examples of positive correlations of ABA level and tolerance, like in sunflower, and switchgrass, which suggest that constitutively high ABA levels in tolerant varieties confer a better ability to cope with an adverse water deficit. On the other hand, some native species from the arid regions display a plastic response to these environmental conditions, showing that the highest ABA levels were found in the temporary sensitive *Poa ligularis,* while the lowest ABA levels were identified in the highly tolerant xerophytic species *Papostypa speciosa* [7]. Besides ABA level, ABA sensitivity is also an important trait for plant survival. Results from wheat and *Arabidopsis* showed that plants with a high drought tolerance showed a significantly higher ABA sensitivity than the drought-sensitive lines [29,30]. Kumar et al. [31] demonstrated that overexpression of OsSta2 gene (*Oryza sativa Salt tolerance activation 2-Dominant*) enhances the tolerance of transgenic rice plants to salt and osmotic stresses and exhibited a hypersensitivity to ABA. This might suggest that drought-sensitive Chinese cabbage needs far more ABA upon drought in comparison to more tolerant species in an attempt to protect against water deficit.

Although SA has been considered as the main stress hormone in biotic stress, the significance of SA has been increasingly recognized in improved plant abiotic stress tolerance [32]. However, the role of SA is still unclear, since some investigators have reported an enhancement of drought tolerance by SA application, whereas others claimed a reduction in the drought tolerance. Generally, the impact of SA is highly dependent on the concentration applied. Thus, experiments with exogenously applied low concentrations of SA were shown to decrease the oxidative stress and enhance the drought tolerance in maize, wheat, tomato, bean, etc. [33]. Furthermore, SA-accumulating mutants of *A. thaliana* (*adr1*, *myb96-1d*, *siz1*, *acd6*, *and cpr5*) exhibited stomatal closure and improved drought tolerance [32,33]. Our results are in agreement with the positive correlations between the SA endogenous level and drought tolerance, since we showed a significant increase of SA in a more tolerant kale upon drought in comparison to the more sensitive white and Chinese cabbages. Gurrurari et al. reported that SA is able to improve the stability of photosynthetic apparatus by positively influencing photosynthetic pigments and protein biosynthesis [34]. The same authors reported that the SA treatment of some plants enhanced F_v_/F_m_ under stress conditions.

There have been many reports on the involvement of JA in drought stress [35]. However, the role of JA in drought stress remains controversial. In some studies, JA has been reported to improve drought tolerance, but in others, it has been reported as a negative agent that causes a notable reduction in growth and yield [36]. The main reason for the controversial data lies in the different experimental setups and the different developmental stages of the investigated plants. The activation of a jasmonate signaling pathway was reported to be involved in improving the drought tolerance in species such as chickpea, rice, *Arabidopsis*, and maize [8,35]. Several reports suggest that the exogenous application of jasmonates ameliorates the response of plants to drought stress. JAs might induce anti-oxidant responses, including ascorbate metabolism, enhance the activity of anti-oxidant enzymes, proline accumulation, chlorophyll levels. Savchenko et al. reported that treatment with the JA precursor 12-OPDA elevated the OPDA levels, and thereby induced stomatal closure and increased the drought tolerance in *A. thaliana* [37]. Under our experimental conditions, JA was significantly increased in more sensitive varieties, while JA-Ile was significantly elevated in all the selected Brassicas in drought. 

#### 3.2.2. Brassinosteroids

Apart from their role in the regulation of plant growth and development, BRs have also been implicated in the control of plant stress responses [38]. Gruszka et al. showed that CS and 24-*epi*BL were accumulated in several barley genotypes in drought conditions [38]. Here, we showed that TY, an early metabolite in the biosynthetic pathway, was significantly increased in kale, while the biologically active forms of BRs, CS and particularly BL were significantly increased only in *B. rapa* upon water deficiency. Recent reports indicate that reduced BL accumulation improves *Arabidopsis*’ tolerance to drought [39]. This finding is in agreement with our results, which showed that drought-tolerant species did not accumulate BL, nor its precursor CS, upon drought. A recent study showed that the AtBAT1 transgenic creeping bentgrass (*Agrostis stolonifera* L.) plants, which reduced the endogenous levels of BRs, displayed drought tolerance as well as delayed senescence [40]. Recent data also showed that BR mutants of barley with lower endogenous BR levels produced less osmoprotectant (proline) [41], which was in agreement with our findings for more tolerant Brassicas. 

#### 3.2.3. Auxins 

Our results showed that drought caused a significant increase of IAA in kale, which is a more tolerant species, while a significant decrease of IAA level was detected in the Chinese cabbage, the most sensitive species (Figure 5). Our results are in agreement with the literature data, suggesting that an increase in the IAA level is apparently linked to growth reduction upon stress and consequently better survival in unfavorable environmental conditions [42]. Results obtained on wheat showed that IAA content of drought tolerant genotype (JM-262) increased by 40.18% while drought sensitive one (LM-2) only by 15.74% upon drought treatment compared with the controls [43]. Transcriptomic analysis confirmed that the tolerant-genotype JM-262 had more upregulated and less downregulated genes that were involved in IAA biosynthesis compared to LM-2. Genetic manipulations in the auxin biosynthetic pathway leading to enhanced endogenous levels of auxin resulted in an improved drought tolerance in *Arabidopsis* [44,45], potato [46], and poplar [47]. Furthermore, exogenously applied IAA to *Arabidopsis* also enhanced the drought tolerance in comparison to untreated plants [45].

#### 3.2.4. Cytokinins

CKs can have both positive and negative effects on drought tolerance, depending on the stress duration or intensity, as well as on the plant species. In the review of Bielach et al. it was reported that enhanced IPT expression (SARK:IPT) and consequently higher endogenous CKs levels caused a delay in the senescence and improved the drought tolerance in many species, such as transgenic cotton, tobacco, rice, peanut, etc. [48]. On the other hand, an analysis of the *Arabidopsis* CKX overexpressor lines and of the *ipt1*, *ipt3*, *ipt5* and *ipt7* mutants revealed that a reduction in cytokinin levels improves the drought stress tolerance [49]. It is obviously hard to generalize about what could be the best strategies for altering the cytokinins profile/level to improve the drought tolerance in plants. Verslues emphasized that preventing a stress-induced decrease in endogenous cytokinin levels in the shoot has a positive effect on the abiotic stress tolerance, largely due to delaying or blocking the senescence [50].

Here we showed that kale, which is a tolerant variety, increased the level of CK ribosides, particularly *c*ZR and iPR, in comparison with other, more sensitive, varieties (the Chinese and white cabbages). In addition, free bases, mostly *c*Z, were significantly increased in all the investigated Brassicas, with the highest increase being in the kale. In contrast, in more sensitive species (the Chinese and white cabbages), CK nucleotids, particularly *c*ZRMP, which are the precursors of active forms, were significantly elevated upon drought. Until now, experimental evidence about the abiotic stress tolerance of plants and *c*Z levels are lacking. Literature data reveal that *c*Z-type CKs tend to accumulate under the particular circumstances associated with limited growth, usually connected to abiotic stresses [51]. Since *c*Z(R) has been approved as less active than *t*Z(R), the function of *c*Z(R) in plants was assumed to be the maintenance of minimal CK activity under growth-limiting conditions, including abiotic stresses. So far, it has been found that the levels of *c*Z(R) were highly up-regulated in tobacco roots in response to drought stress [52]. After re-watering, *c*Zs were down-regulated, with a simultaneous increase of *t*Z-type CKs in tobacco, which is in agreement with our results for all Brassicas. Drought conditions caused an increase in the CKs storage forms *O*-glucosides only in *B. rapa*, while *N*-glucosides, among which were mostly levels of iP7G and *c*Z7G, increased in the kale. After recovery, the levels of *N*-glucosides were additionally significantly high in the white cabbage and the kale in comparison with the corresponding controls, in parallel with the decrease of *c*Z. Li et al. showed that *Arabidopsis* plants overexpressing UGT76C2, the enzyme that catalyzes the *N*-glycosylation of all classic cytokinins, were tolerant to drought stress as adult plants, while the knockout *ugt76c2* mutants showed the opposite response, indicating the protective role of *N*-glucosides against drought [53].

### 3.3. Hormonal Cross-Talk in the Drought Response

Drought tolerance is genetically determined, and it is the result of complex processes and networks of numbers of biochemical and molecular players. The stress hormone ABA is the most prominent drought stress marker, and increased significantly in all three *Brassica* species. In addition to a moderate ABA increase, the most tolerant kale was characterized by a significant increase in JA-Ile, SA, IAA, the brassinosteroid early precursor TY, and the cytokinins iP(R) and *c*Z(R). On the other hand, the most sensitive species, *B. rapa* was accompanied with the highest increase of ABA among the investigated crops, together with a significant increase of jasmonates (JA and JA-Ile), the brassinosteroids active forms CS and BL, the cytokinins precursor *c*ZRMP and a moderate increase of *c*Z and *O*-glucosides. Considering drought tolerance, the white cabbage was moderately tolerant to drought, characterized by increased ABA, jasmonates and moderate *c*Z. Investigating the hormonal crosstalk in rice upon drought, Deb et al. concluded that “multiple hormones may modulate hormone biosynthesis pathways through a complex regulatory network, where biosynthesis of one hormone is affected by several other contributing hormones” [38]. They reported that ABA usually acts positively with JA and BRs, while it might be antagonistic to SA. It is furthermore postulated that ABA acts as an antagonist to CKs in various environmental stress responses. The results of a comprehensive study of rice response to drought stress showed decreased levels of CKs, gibberellin GA1, and IAA, while levels of ABA and JA increased [54]. It was furthermore interesting that observed changes in CKs and IAA in the rice under stress conditions were not caused by transcriptional regulation of the genes responsible for their biosynthesis and catabolism. The authors speculated that drought stress affects the distribution or degradation of CKs and IAA molecules. Ha et al. [55] summarized in their review that a reduction in CK content usually leads to hypersensitivity to ABA and the upregulation of stress- and/or ABA-responsive genes, whereas an increase in CK content led to the downregulation of ABA and stress-responsive genes. In addition, CKs were reported to interact negatively with JA and BR. Janeczko et al. reported that barley mutants with impaired BR production were characterized by a reduced height and developmental retardation and showed the reduced production of ABA and cytokinins, but not auxins under drought conditions [41]. ABA and auxins usually act antagonistically. An interesting enhanced drought tolerance through an auxin- and ABA-mediated signaling response was reported recently in an overexpressing line (AtEDT1/HDG11) of Chinese kale that exhibited auxin-overproduction phenotypes (long hypocotyls, tall stems, more root hairs, and a larger root-system architecture) and a significantly reduced stomatal density [56]. More importantly, AtEDT1/HDG11-overexpression leads to ABA hypersensitivity. CKs have been, on the other hand, a positive regulator of auxin biosynthesis, and it was proposed that both CKs and IAA signaling are involved in drought tolerance [8,48].

### 3.4. Correlations between Phytohormones, Oxidative Stress and Photosynthesis

Based on the results, a few clear correlations between phytohormones, photosynthesis and oxidative stress in selected Brassicas can be concluded. Accordingly, the photosynthetic parameters (PI_ABS_ and F_v_/F_m_) were negatively correlated with ABA, JA, and BRs, while SA and CKs show more positive effects. ABA plays an important role in regulating plants’ water status and growth by inducing stomatal closure and regulating ABA-responsive genes under abiotic stress conditions [27]. The decreased photosynthetic activity observed, particularly in *B. rapa*, can be explained by the significant increase in the ABA level under these conditions. On the other hand, the highest increase of SA was found for kale in which the photosynthetic parameters were less affected under drought conditions. It was previously reported that SA may act protectively on the photosynthesis during stress by increasing the photosynthetic rate, carbon fixation, transpiration, stomatal conductance and anti-oxidant activity in many plant species [34,57,58]. CKs, particularly ribosides and free bases, were mostly increased in the most tolerant kale and they may act protectively on the photosynthesis and general plant vitality. It has been well documented so far that CKs improve tolerance largely due to delaying or blocking the senescence [50,55]. ABA, JAs and BRs also strongly affect the synthesis and accumulation of osmoprotectants such as proline [8,35,59], which is in agreement with the results obtained here. 

A better understanding of hormonal profiles and their crosstalk under stress conditions as well as their correlations with physiological and biochemical parameters in stress responses might be crucial for biotechnological strategies in stabilizing agriculture yields under unfavorable environmental conditions.

## 4. Materials and Methods 

### 4.1. Plant Material

Seeds of Chinese cabbage (*Brassica rapa* L. ssp. *pekinensis* (Lour.) Hanelt cv. Cantonner Witkrop) were purchased from ISP International Seed Processing GmbH, Quedlinburg, Germany, while seeds of white cabbage (*Brassica oleracea* var. *capitata* cv. Varaždinski) and kale (*Brassica oleracea* var. *acephala* cv. IJK9) were obtained from Agricultural Advisory Service from Varaždin Region, Croatia, and Institute for Adriatic Crops and Karst Reclamation, Split, Croatia, respectively. For root-growth bioassay, seeds were germinated on 1% agar plates and one-day old seedlings (with root length approximately 1 cm) were placed to agar plates supplemented with mannitol in the range of concentration 0–400 mM, for 24 h. Root growth of treated seedlings were reported as % of untreated controls. For drought experiments, seeds were germinated on 1% agar plates and few-days old seedlings were potted into soil, each seedling in individual plastic pot, and growing for 4 weeks with regular watering, in a growth chamber at 21 °C and photoperiod 16/8 h (light/dark). At the stage of 4 weeks old, one group of plants was subjected to drought until their leaves reached RWC of approximately 45%. One group of plants was then recovered by watering to RWC of approximately 85%. Three groups of plant material were analyzed: plants subjected to drought, plants subjected to recovery for 24 h after drought, and corresponding controls. 

Photosynthesis measurements were performed in vivo using 7 individual plants per treatment. All other measurements were performed on four replicates if not reported otherwise. One replicate considers 5 individual plants, regularly watered (controls) or treated (drought and recovery). Biochemical analyses were performed using harvested leaves that had been stored at −80 °C, and analytical measurements of plant hormone levels were performed using lyophilized plant material. 

### 4.2. Photosynthetic Parameters Measurements

The photosynthetic efficiency was determined by performing chlorophyll *a* fluorescence measurements in vivo using a Plant Efficiency Analyzer (PEA, Hansatech, Norfolk UK). Measurements were performed using 7 plants per treatment, and the plants were dark-adapted for about 30 min before the measurements. Chlorophyll fluorescence transients (OJIP) were induced by applying a pulse of saturating red light with maximum intensity at 650 nm and a photon flux of 3000 mmol m^−2^ s^−1^. Changes in fluorescence were measured over 1 s and the obtained data were used to calculate the maximum quantum yield of PS II (F_v_/F_m_) and the Performance index (PI_ABS_) [60].

### 4.3. Biochemical Stress Markers 

Leaves of 5 plants per treatment were subjected to the leaf relative water content measurement (RWC). Fresh weight, turgid weight (24 h of soaking leaves in water) and dry weight (24 h at 80 °C) were recorded, respectively, and RWC was estimated as the percentage according equation: (fresh weight-dry weight)/(turgid weight-dry weight) × 100. 

The seedlings’ lipid peroxidation, protein oxidation, proline, and ascorbate (AA) levels were determined using the previously reported methods [61]. In brief, lipid peroxidation was determined by spectrophotometry using the malondialdehyde (MDA) method. Absorbances at 532 and 600 nm were measured, and the resulting values were used to estimate the sample’s MDA content based on an assumed extinction coefficient of 155 mM^−1^ cm^−1^.

The amount of protein oxidation was estimated by the reaction of carbonyl groups with 2,4-dinitrophenylhydrazine (Sigma-Aldrich, Saint Louis, MO, USA). The carbonyl content was calculated by absorbance at 370 nm, using an extinction coefficient of 22 mM^−1^ cm^−1^ and expressed as nanomoles of carbonyl per milligram of protein.

Free proline levels were measured spectrophotometrically at 520 nm using ninhydrin. A calibration curve was constructed using L-proline as a standard and used to relate the measured absorbances to proline concentrations in nmol proline per gram of fresh weight.

Ascorbate levels were determined by spectrophotometry at 530 nm based on a calibration curve created using sodium ascorbate as the standard. All absorbance measurements including four replicates of each assay were performed on spectrophotometer (Analytik Jena Specord 40, Analytik Jena, Jena, Germany).

### 4.4. Antioxidant Enzymes Assays

Activities of superoxide dismutase (SOD; EC 1.15.1.1), ascorbate peroxidase (APX; EC 1.11.1.11), and catalases (CAT; EC 1.11.1.6) were determined using the previously reported methods [61]. All absorbance measurements, including four replicates of each assay, were performed on spectrophotometer (Analytik Jena Specord 40, Analytik Jena, Jena, Germany).

### 4.5. Plant Hormones Identification and Quantification

#### 4.5.1. Auxin and Stress Hormones 

Auxin (indole-3-acetic acid, IAA) and stress-related hormones such as jasmonates (jasmonic acid, JA and jasmonoyl-isoleucine, JA-Ile), SA and ABA were purified as described previously with minor modifications [62]. Briefly, lyophilized samples (5 mg DW) were homogenized with a MixerMill (Retsch GmbH, Haan, Germany), extracted in 1 mL 50 mM sodium phosphate buffer (pH 7.0) containing 1% sodium diethyldithiocarbamate and stable isotope-labelled internal standards (5 pmol each of [^13^C_6_]IAA, [^2^H_4_]SA, [^2^H_6_]JA, [^2^H_2_]JA-Ile and [^2^H_6_]ABA; OlChemIm) and then alkalized by adding 1 mL of 5% NH_4_OH/H_2_O (*v*/*v*). The resulting solution was applied to a mixed-mode anion exchange columns (Oasis^®^ MAX column, 1 cc/30 mg, Waters, Milford, MA, USA) conditioned with 100% MeOH, equilibrated with 5% NH_4_OH/H_2_O (*v*/*v*), and washed with H_2_O. The column was then washed with 2 mL 5% NH_4_OH followed by 2 mL 100% MeOH, and the acidic phytohormones were eluted using 2 mL 2% HCOOH in 100% MeOH (*v*/*v*). The samples were evaporated to dryness under a stream of nitrogen and stored in a freezer at −20 °C until analysis. 

#### 4.5.2. Cytokinins

Cytokinins were analyzed according to Antoniadi et al. [63]. Briefly, samples (2.5 mg DW) were homogenized and extracted in 1 mL of mL of modified Bieleski buffer (60% MeOH, 10% HCOOH and 30% H_2_O) together with a cocktail of stable isotope-labeled internal standards (0.25 pmol of CK bases, ribosides, *N*-glucosides, and 0.5 pmol of CK *O*-glucosides, nucleotides per sample added; OlChemIm). The extracts were purified using the Oasis MCX column (30 mg/1 mL, Waters Milford, MA, USA) conditioned with 1 mL each of 100% MeOH and H_2_O, equilibrated sequentially with 1 mL of 50% (*v*/*v*) nitric acid, 1 mL of H_2_O, and 1 mL of 1M HCOOH. After sample application onto an MCX column, unretained compounds were removed by a wash step using 1 mL of 1M HCOOH and 1 mL 100% MeOH, preconcentrated analytes were eluted by two-step elution using 1 mL of 0.35M NH_4_OH aqueous solution and 2 mL of 0.35M NH_4_OH in 60% (*v*/*v*) MeOH solution. The eluates were then evaporated to dryness in vacuo and stored at −20 °C.

#### 4.5.3. Brassinosteroids

Samples were analyzed for BR contents as described previously with a few modifications [64]. Briefly, 40 mg of dry tissue samples was homogenized to a fine consistency using 3-mm zirconium oxide beads and an MM 301 vibration mill at a frequency of 30 Hz for 3 min (Retsch, Haan, Germany). The samples were then extracted overnight with stirring at 4 °C using a benchtop laboratory rotator (Stuart SB3; Bibby Scientific, Cole-Parmer, Staffordshire, UK) after the addition of 1 mL of ice-cold 60% acetonitrile and 30 pmol of [^2^H_3_]BL, [^2^H_3_]CS, [^2^H_3_]TY, [^2^H_3_]24-*epi*BL, [^2^H_3_]24-epicastasterone, [^2^H_3_]28-norbrassinolide, and [^2^H_3_]28-norcastasterone as internal standards (OlChemIm, Olomouc, Czech Republic). The samples were further centrifuged, purified on polyamide SPE columns (Supelco, Sigma-Aldrich), then evaporated to dryness in vacuo and stored at −20 °C.

#### 4.5.4. Instrument Set up

The evaporated samples prepared for plant hormone analyses were dissolved in 40 µL of the mobile phase prior to determination using UHPLC-MS/MS. Metabolites were analyzed by an Acquity UPLC System (Waters, Milford, MA, USA) coupled by to a triple quadrupole mass spectrometer Xevo™ TQ-S MS (Waters, MS Technologies, Manchester, UK) using stable isotope-labelled internal standards as references and calculated by the isotope dilution method. Instrument settings were as published previously for profiling of auxins [65], stress-related phytohormones [62], cytokinins [66], and brassinosteroids [64]. Four independent replicate analyses were performed for each hormone group if not reported otherwise. 

### 4.6. Statistical Analysis

The results of each analysis were compared using analysis of variance (ANOVA) with Tukey’s HSD Post Hoc test, as implemented in the XLSTAT software package; differences between exposure treatments and the corresponding controls were considered statistically significant if *p* < 0.05. Each reported data point represents the average of four replicates (*n* = 4) unless stated otherwise. For adequate comparison of varieties that exhibited different drought tolerance, all data were normalized to set up all controls to value 1. Original data are available in Tables S1–S7.

### 4.7. Principal Component Analysis (PCA)

For multifactorial comparison, principal component analyses (PCA) was used to display the correlation among the various physiological, biochemical, and hormonal parameters and their relationship with drought treatment. PCA was performed based on the correlation matrix of average values of traits after standardization (autoscaling). The missing values of phytohormone metabolites in some plants subjected to drought, recovered for 24 h after drought, and corresponding controls were imputed with two-thirds of their respective limit of detections [67]. Pearson coefficients (n) were calculated to evaluate linear correlation among variable. Procedures were performed using Microsoft Office Excel 2010 upgraded with XLSTAT (https://www.xlstat.com/en/, ver. 2017.01.40777).

## 5. Conclusions

Based on the reported results for three selected *Brassica* crops, we highlighted kale (*Brassica oleracea* var. *acephala*) as the most drought-tolerant variety, then white cabbage (*B. oleracea* var. *capitata*) as moderately tolerant, and finally Chinese cabbage (*B. rapa*) as the most sensitive one. Furthermore, our results showed correlations between drought tolerance and certain phytohormones changes that might be responsible, in part, for the tolerance/sensitivity of the investigated species/varieties. In brief, drought tolerance in kale correlated mostly with increased level of SA, ABA, IAA, cytokinins iP(R) and *c*Z(R), and TY, the precursor of active brassinosteroids. On the other hand, the drought sensitivity of the Chinese cabbage correlated mostly with a significant increase of ABA, jasmonates and the active brassinosteroids CS and BL. The moderately tolerant white cabbage positioned between the kale and the Chinese cabbage showed more similarity in the phytohormone patterns with the kale. A re-watering treatment for 24 h caused phytohormone changes towards the control conditions, mostly in the kale, while the Chinese and white cabbages showed a slower tendency for recovery. Based on the obtained results we can conclude that the drought tolerance of species/varieties from the *Brassica* genus are mediated by increased endogenous levels of IAA, CKs, ABA and SA, while the synthesis of active BRs is inhibited. These phytohormones and their metabolic pathways can be considered as important metabolic targets for producing drought-tolerant *Brassica* plants. However, we are aware that the hormonal cross-talks in drought stress are very complex, and additional research at the molecular level is necessary to shed light on the hormonal players and the interactions responsible for drought tolerance in Brassicaceae.

## Figures and Tables

**Table 1:** The photochemical parameters measured in *Brassica* crops upon drought and recovery: the performance index (PI_ABS_) and maximum quantum yield of PS II (F_v_/F_m_). Controls are normalized to the value 1. Same letters present non-significant values at *p* < 0.05. Data are mean ± SD, *n* = 7. Raw data are presented in Supplementary Table S1.

Cultivar	Treatment	PI_ABS_	F_v_/F_m_
*B. rapa* ssp. *pekinesis*	Control	1.000 ± 0.106 ^ab^	1.000 ± 0.012 ^ab^
	Drought	0.438 ± 0.211 ^c^	0.894 ± 0.059 ^c^
	Recovery	0.949 ± 0.307 ^ab^	0.966 ± 0.039 ^ab^
*B. oleracea* var. *capitata*	Control	1.000 ±0.048 ^ab^	1.000 ± 0.005 ^ab^
	Drought	0.437 ±0.110 ^c^	0.955 ± 0.012 ^b^
	Recovery	0.601 ± 0.136 ^bc^	0.977 ± 0.012 ^ab^
*B. oleracea* var. *acephala*	Control	1.000 ± 0.328 ^ab^	1.000 ± 0.020 ^ab^
	Drought	1.239 ± 0.456 ^a^	1.007 ± 0.017 ^a^
	Recovery	1.390 ± 0.406 ^a^	1.014 ± 0.017 ^a^

## Supplementary Materials

Supplementary materials can be found at http://www.mdpi.com/1422-0067/19/10/2866/s1. Table S1: Photosynthetic parameters, maximum quantum yield of PS II (F_v_/F_m_) and the performance index (PI_ABS_), measured in *Brassica rapa*, *B. oleracea* var. *capitata* and *B. oleracea* var. *acephal*a upon drought and recovery in comparison to corresponding controls; Table S2: Endogenous levels of proline (L-PRO), ascorbic acid (AA), malondialdehyde (MDA) (nmol/g FW) and carbonyl groups (CO) (nmol/mg proteins) measured in *Brassica* crops upon drought and recovery in comparison to the corresponding controls; Table S3: Activity of antioxidant enzymes: catalase (CAT), ascorbate peroxidase (APX) and superoxide dismutase (SOD) (U/mg proteins) measured in *Brassica* crops upon drought and recovery; Table S4: Endogenous levels of abscisic acid (ABA), salicylic acid (SA), jasmonic acid (JA), jasmonoyl-isoleucine (JA-Ile) and indole-3-acetic acid (IAA) (pmol/g DW) in *Brassica* crops upon drought and recovery in comparison to the corresponding control; Table S5: Endogenous levels of brassinosteroids typhasterol (TY), castasterone (CS) and brassinolide (BL) (pg/g DW) in *Brassica* crops upon drought and recovery in comparison to the corresponding controls; Table S6: Endogenous total cytokinin levels (pmol/g DW) in *Brassica* crops upon drought and recovery in comparison to the corresponding control; Table S7: Endogenous levels of individual cytokinins (pmol/g DW) in *Brassica* crops upon drought and recovery in comparison to the corresponding control; Table S8: Relative total cytokinins levels in *Brassica* crops upon drought and recovery in comparison to the corresponding control; Table S9: Relative total and individual cytokinins in *Brassica* crops under drought and recovery in comparison to the corresponding control; Table S10: Correlation matrix (Pearson (n)); Table S11: Eigenvalues; Table S12: Factor loadings and correlations between variables and factors; Table S13: Factor scores; Table S14: Squared cosines of the variables.

## Abbreviations

AA	ascorbic acid
ABA	abscisic acid
APX	ascorbate peroxidase
BL	Brassinolide
BRs	Brassinosteroids
CAT	Catalase
CKs	Cytokinis
CS	Castasterone
*c*Z	*cis*-zeatin
*c*Z9G	*cis*-zeatin 9-glucoside
*c*ZOG	*cis*-zeatin O-glucoside
*c*ZR	*cis*-zeatin riboside
*c*ZRMP	*cis*-zeatin riboside-5′-monophosphate
*c*ZROG	*cis*-zeatin riboside O-glucoside
IAA	indole-3-acetic acid
iP	N^6^-isopentenyladenine
iP9G	N^6^-isopentenyladenine 9-glucoside
iPR	N^6^-isopentenyladenosine
ISCK	isoprenoid cytokinins
JA	jasmonic acid
JA-Ile	jasmonoyl-isoleucine
JAs	Jasmonates
MDA	malondialdehyde
PCA	principal component analysis
PS II	photosystem II
SA	salicylic acid
SOD	superoxide dismutase
TY	Typhasterol
*t*Z	*trans*-zeatin
UHPLC-MS/MS	ultra-high performance liquid chromatography–tandem mass spectrometry
